# Heat Stress in Dairy Cows: Impacts, Identification, and Mitigation Strategies—A Review

**DOI:** 10.3390/ani15020249

**Published:** 2025-01-17

**Authors:** Charles Paranhos Oliveira, Fernanda Campos de Sousa, Alex Lopes da Silva, Érica Beatriz Schultz, Roger Iván Valderrama Londoño, Pedro Antônio Reinoso de Souza

**Affiliations:** 1 Departament of Agricultural Engineering, Universidade Federal de Viçosa, Viçosa 36570-900, Brazil; pedro.antonio.souza@ufv.br; 2 Departament of Animal Science, Universidade Federal de Viçosa, Viçosa 36570-900, Brazil; alex.lopes@ufv.br (A.L.d.S.); erica.schultz@ufv.br (É.B.S.); 3 Institute of Biology, Faculty of Exact and Natural Sciences, Universidad de Antioquia, Medellín 1226, Colombia; roger.valderrama@udea.edu.co

**Keywords:** ambience, machine learning, animal environment, animal welfare, homeothermy, dairy farming

## Abstract

Heat stress in animals affects productivity, health, and reproduction, with particularly pronounced effects in dairy cows. Identifying heat stress requires understanding both physiological and environmental indicators, such as increased heart rate, respiratory rate, and rectal temperature, which reflect the animal’s thermal condition within its environment. Thermoregulation in cows involves behavioral and physiological adjustments to maintain homeothermy, aiming to stabilize their internal thermal state. To assess the thermal condition of animals, machine learning models have been developed, leveraging both environmental and physiological indicators for more accurate stress detection. Among the various indices of thermal environment, the Temperature and Humidity Index (THI) is the most widely used. Cooling strategies for animals and their environments are essential to mitigate the effects of heat stress. One effective approach involves the use of evaporative adiabatic cooling combined with forced ventilation systems in feeding alleys and pre-milking holding areas. This method enhances evaporative exchanges and facilitates heat dissipation between the animal and its surroundings, thereby alleviating heat stress and improving both the welfare and productivity of dairy cows.

## Simple Summary

Heat stress in dairy cows is a serious issue that can negatively impact milk production, health, and reproduction. Cows attempt to adapt to heat by altering their behavior to maintain a stable body temperature. When cows are in thermally challenging environments, it is crucial to identify signs of heat stress. Indicators of heat stress in cows include an increased heart rate, faster breathing, and elevated body temperature. To assist in identifying heat stress, scientists are developing technologies that monitor environmental and health data. One tool used to assess the thermal condition of the environment in relation to animals is the Temperature and Humidity Index (THI). When heat stress is detected, it is essential to cool the cows and improve their environmental conditions. Effective methods include using water sprinklers and fans to enhance heat exchange between the animals and their surroundings. These measures help cool the cows, improve their welfare, and result in healthier animals with higher milk production.

## 1. Introduction

In countries with a predominantly tropical climate, characterized by high air temperatures for most of the year, heat stress can compromise the health and welfare of production animals. Given this scenario, dairy production faces a significant challenge, as the animals that have been genetically selected over the years for high milk productivity are predominantly European breeds, especially Holstein cows. Cows are homeothermic animals, meaning they have the ability to maintain a stable core body temperature within a relatively narrow range, even when exposed to fluctuations in environmental temperature or activity levels [1]. However, Holstein cows, which are native to temperate climates, experience performance limitations when exposed to regions with high ambient temperatures.

Numerous studies have been conducted to identify the onset of heat stress in dairy cows [2,3,4]. These studies reveal that exposure to elevated environmental temperatures induces physiological and behavioral changes aimed at maintaining homeothermy. In this sense, there is an increase in respiratory rate, a reduction in food consumption, an increase in heart rate, an increase in water consumption, and a tendency for animals to seek out shaded areas, among others [3,5]. These modifications at the physiological level represent an increased energy expenditure for the animals, and, when in thermally unfavorable environments, there is a reduction in production, a decrease in estrus intensity and fertility, as well as various health damages and increased susceptibility to disease [6,7,8,9].

To assess the level of comfort an environment provides, the Temperature and Humidity Index (THI) has traditionally been used [10]. However, this index does not account for animal-related factors needed to evaluate environmental conditions comprehensively. An alternative approach is to leverage machine learning tools to identify and predict heat stress in dairy cows [11,12,13]. By analyzing large datasets from sensors that measure variables such as body temperature, respiratory rate, heart rate, and activity, machine learning techniques can be trained to detect patterns and subtle indicators of heat stress. These algorithms can accurately classify and predict changes in animal conditions, enabling quicker and more effective interventions. By integrating real-time monitoring systems with predictive models, producers can proactively adjust environmental and management conditions before heat stress adversely affects animal health and productivity [14]. Machine learning, by combining physiological, behavioral, and environmental data, emerges as a promising tool to enhance control over the thermal comfort conditions of dairy cows.

In this context, strategies to reduce thermal stress in dairy cows have been developed and implemented in various regions worldwide. In temperate climate countries, these strategies are primarily applied during the summer, when environmental temperatures are relatively high [1,15]. In tropical and subtropical countries, however, these strategies are adopted throughout most of the year, due to the low variation in environmental temperatures, which remain relatively high for the majority of the year [1,16,17,18]. As a result, dairy cow farming in intensive production systems is typically carried out in facilities with roofs to reduce the thermal radiation load on the animals and ventilation systems to promote air renewal and facilitate thermal exchange [1,5,19,20]. Along with ventilation systems, cooling systems are commonly employed to lower air temperature, such as evaporative panels in tunnel-ventilation systems and sprinklers or misters in facilities with positive pressure ventilation systems [1,19,20].

Given this context, the objective of this literature review was to develop an approach to the main physiological indicators of heat stress in intensively managed dairy cattle and its impacts on milk production, reproduction, and animal health. Additionally, cooling strategies for these animals are presented, taking into consideration the different constructive typologies of facilities designed for animal housing in dairy cattle operations.

## 2. Thermoregulation

**Figure 1:**
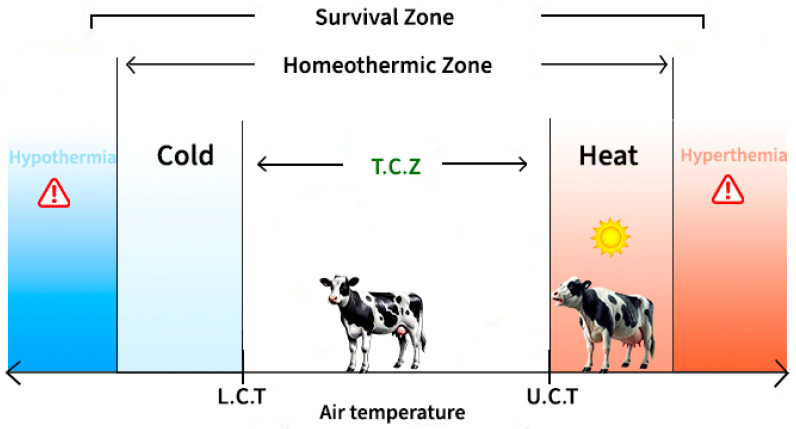
Schematic representation of animal comfort, homeothermic, and survival zones. Where T.C.Z. é the thermal comfort zone, L.C.T is the lower critical temperature, and U.C.T. is the upper critical temperature.

Dairy cattle are homeothermic animals, meaning they have the ability to maintain their core body temperature within relatively narrow limits, regardless of variations in ambient temperature or activity level [1,15]. Heat stress in animals is characterized by elevated ambient temperatures above their thermal comfort zone. When an animal is within the thermoneutral zone, heat production remains stable without energy expenditure for activating thermoregulatory mechanisms, allowing the animal to express its maximum productive potential [16,21]. When ambient temperatures exceed the critical threshold, there is an increase in the animal’s heat production as it activates thermoregulatory mechanisms to dissipate heat to the environment and maintain homeothermy and stable core body temperature [21,22,23,24,25] (Figure 1).

Heat exchange between the animal and the environment occurs bidirectionally through two mechanisms: sensible and latent heat transfer. In dairy cattle and all homeothermic animals, sensible heat exchange occurs through convection, conduction, and radiation [1,26]. For sensitive exchanges to take place, a temperature gradient is required between the animal and its environment [15]. Thus, at environmental temperatures slightly above the upper critical temperature, a common behavior is for the animal to stand up longer in order to increase convective exchange with the environment [3,22,26]. When the ambient temperature rises significantly above the upper critical temperature, latent heat exchange mechanisms are activated, becoming the predominant form of energy transfer [1,27,28]. Latent heat exchange occurs through evaporation and condensation processes, via increased sweating and panting [15]. For these exchanges to occur, a vapor pressure gradient must be present, with relative humidity playing a critical role in these forms of heat transfer. In environments with high temperatures and elevated relative humidity, the animal becomes unable to dissipate heat effectively, exacerbating heat stress [1,26]. This can ultimately result in the animal collapsing and succumbing to hyperthermia [7].

Zhou et al. (2022) [28] investigated the effects of heat stress on sensible and latent heat losses in Holstein cows using a climate-controlled respirometry chamber. These authors observed that when the air temperature was below 20 °C, heat exchange was evenly distributed, with approximately 50% occurring through latent pathways and the other 50% through sensible pathways. When air temperature exceeded 28 °C, evaporation became the primary heat loss mechanism, representing approximately 70–80% of total animal heat loss. Those authors also noted that respiratory heat exchange accounted for 20–30% of heat losses, and when temperature increased from 16 to 32 °C, respiratory heat exchange increased by 34%. In dairy cattle, panting indicates severe heat stress; other behavioral indicators of thermal stress include increased standing time, elevated water consumption, and reduced feed intake.

In animals, thermal stress is diagnosed through the hypothalamic–pituitary axis, which acts on the thyroid and reduces the levels of triiodothyronine (T3) and thyroxine (T4) hormones to decrease animal metabolism. As a consequence of reduced metabolism, the animal decreases feed intake, resulting in fewer available nutrients in the organism [22]. Another physiological modification is vasodilation, where increased blood flow occurs from the body core toward the skin [5]. This means that animals experiencing heat stress exhibit elevated surface temperatures to enhance heat dissipation into the environment, primarily through convection and evaporation.

## 3. Physiological Aspects of Heat Stress in Dairy Cows

Heat stress in dairy cattle has the potential to reduce milk production, while also having significant impacts on reproduction and health. Determining the point at which an animal experiences thermal stress is crucial for optimizing facility management and thermal conditioning systems, and consequently, for the success of the production system. To determine the thermal environmental conditions for the animal, indices and thermoneutrality ranges based on environmental variables have been developed, such as the Temperature and Humidity Index (THI) [10].

Over the years, the heat stress threshold has been decreasing due to genetic improvement, requiring increased attention to production systems to ensure a comfortable environment for animals. Currently, an acceptable THI limit for high-producing dairy cows (above 35 kg·day^−1^) is up to 68 [20]. However, since THI is based on environmental variables (air temperature and relative humidity), the perception of stress at the animal level may be compromised. An alternative to this problem is using the animal itself as a stress indicator. Several approaches have been adopted at the research level to quantify and establish reliable relationships regarding the onset of thermal stress in animals [13,23,29]. Animal indicators such as respiratory rate, core body temperature, and decreased milk production have proven to be reliable indicators of the heat load perceived by the animal [3,13]. Additionally, decreased milk production serves as an indicator of heat stress in cows [3,20]. However, reduced milk production is not an instantaneous response, as there is a lag between the onset and duration of thermal stress and the consequent decline in production [20]. Therefore, the animal’s physiological indicators appear to be the best markers for determining the moment of stress. Variations in body temperature and respiratory rate are the first signs that the thermoneutrality state has been disturbed and the cow is experiencing thermal stress [3].

Animal body temperature can be verified through various methods, including internal measurements: rectal, vaginal, ruminal, and tympanic temperatures, and external measurements: surface temperature, such as infrared thermography, as reported in various studies [4,13,30]. In a study with lactating Holstein cows housed for four days in climate chambers, under heat stress conditions with THI varying between 74 and 84 and in thermoneutral conditions with THI between 55 and 61, Garner et al. (2017) [30] observed that rectal and vaginal temperatures were significantly higher in heat-stressed cows. In the heat stress treatment, values of 40 °C were recorded for both rectal and vaginal temperatures, while in thermoneutral conditions, values were 38.5 °C for rectal temperature and 38.8 °C for vaginal temperature. In the same study, the authors observed differences in udder surface temperature, recording 39.8 °C and 35.2 °C for cows housed in heat stress and thermoneutral conditions, respectively. The effects of seasonal variation on rectal temperature in Holstein cows were observed by Rejeb et al. (2016) [4] who conducted research during summer and spring in Tunisia, calculating an average THI of 83.3 for summer and 65.6 for spring, with rectal temperature values of 39.2 °C for summer and 38.2 °C for spring.

Respiratory rate is an efficient physiological indicator for assessing thermal stress in dairy cattle. This mechanism is activated instantaneously in response to heat stress stimuli, as panting is a highly efficient pathway for latent body heat dissipation. In a Serbian study, Vujanac et al. (2010) [31] observed that high-producing cows in early lactation, under heat stress conditions with THI above 70, exhibited respiratory rates varying from 51.4 to 77.89 breaths/min throughout the day; whereas in non-heat stress conditions, with THI below 70, respiratory rates ranged from 46.8 to 51.9 breaths/min. Similar results were observed by Rejeb et al. (2016) [4], who reported respiratory rates of 79.4 breaths/min during summer with THI of 83.3 and 43.9 breaths/min during spring with THI of 65.6. The threshold respiratory rate considered comfortable is below 60 breaths/min, with values between 60 and 80 breaths/min characterized as alert indicators and above 80 indicating dangerous conditions [13]. Cooling is a viable alternative for alleviating thermal stress in dairy cattle. Research conducted in Israel demonstrated that multiparous Holstein cows under thermal stress conditions, with THI above 68, when cooled eight times daily with sprinklers and fans, exhibited a respiratory rate of 60.2 breaths/min, while those cooled three times daily showed a respiratory rate of 73.1 breaths/min [32], emphasizing the importance of cooling methods for dairy cattle.

Heat stress in dairy cows not only affects the physical well-being of the animals, but also has direct consequences for productivity, health, and reproduction. Continuous exposure to unfavorable thermal conditions compromises the reproductive efficiency of cows, reducing fertility and increasing the incidence of abortions [9,33]. In addition, the impacts on milk production are immediate, with reduced yield especially during the most critical periods of heat [30]. Physiological indicators, such as increased respiratory rate and elevated body temperature, demonstrate the onset of heat stress, which reinforces the need for appropriate management [3]. The adoption of cooling measures and the creation of thermally favorable environments are essential to mitigate these effects, in order to ensure the general health of the animals and optimize production and reproduction.

## 4. Heat Stress and Productivity

The reduction in milk production as a consequence of heat stress episodes is widely reported in the literature [4,30,34]. According to Bernabucci et al. (2010) [35], heat stress affects animals’ metabolic and physiological acclimation, reducing milk production both directly, by causing hyperthermia, and indirectly, through decreased dry matter intake and changes in animal behavior. In a climate chamber experiment with multiparous Holstein cows over four days, comparing thermoneutral conditions with heat stress conditions, Garner et al. (2017) [30] observed that milk production was significantly lower for cows housed in heat stress chambers on the fourth day, with production of 7.9 kg/day under heat stress conditions compared to 17 kg/day under thermoneutral conditions. The effects of seasonality due to climate variations also directly influence milk production. Rejeb et al. (2016) [4] reported a reduction of 5.6 kg/day in cows during spring and summer.

Lactating cows are more susceptible to heat stress compared to dry cows. This is attributed to milk production and increased metabolic activity [8]. A common behavior observed in homeothermic animals under heat stress conditions is reduced feed intake, aiming to lower metabolism and, consequently, decrease body heat production. In lactating cows, the reduction in dry matter intake leads to decreased availability of nutrients required for milk synthesis. However, the reduction in milk production in cows under heat stress cannot be attributed solely to reduced dry matter intake. Wheelock et al. (2010) [34] evaluated production parameters and metabolic variables in 22 multiparous Holstein cows subjected to three different treatments: thermoneutral conditions with ad libitum feeding for seven days (P1), heat stress conditions with ad libitum feeding or pair-feeding under thermoneutral conditions (P2), and heat stress conditions with ad libitum feeding supplemented with recombinant bovine somatotropin (rbST) for seven days (P3). These authors observed that dry matter intake decreased by approximately 30% in cows exposed to heat stress. Milk production declined by 9.6 kg during heat stress and 4.8 kg under pair-feeding conditions. Based on these results, the authors concluded that dry matter intake accounted for 50% of the reduction in milk production during thermal stress, with the remaining decrease largely attributed to potential changes in the post-absorptive metabolism of nutrients.

Typically, a reduction in milk production is observed in the days following exposure to heat stress [36]. This delay in the milk production response may range from 24 to 48 h after a persistent heat stress episode lasting four days [8]. Although reduced milk production is a response to thermal stress in dairy cows, its use as an indicator suggests that the cow has already experienced a prior period of heat stress, as it reflects the outcome of the stressor stimulus. Given this, continuous monitoring of the production environment is essential to facilitate the implementation of cooling strategies, ensuring a thermally favorable environment for the animals without compromising milk production.

## 5. Heat Stress and Reproduction

Heat stress in dairy cows directly impacts their reproductive performance, which is multifaceted and involves physiological and behavioral responses. The hypothalamus–pituitary–ovary axis is directly affected by the stress stimulus. Following exposure to stress, there is an increase in plasma follicle-stimulating hormone (FSH) concentration, a decrease in estradiol production—compromising the duration and intensity of estrus—and suppression of luteinizing hormone (LH) secretion [9]. During periods of thermal stress in dairy cows, reduced LH secretion can impair the functional formation of the corpus luteum [9]. Additionally, there is a decrease in blood progesterone levels. These factors indicate reduced embryo viability and an increase in early embryonic mortality.

Elevated ambient temperatures above the thermoneutral zone in dairy cows directly affect ovarian follicles, causing damage to oocytes. Gendelman et al. (2010) [33] studied oocytes collected from Holstein cows during the summer and observed reduced development, as well as low progression of embryos to the blastocyst stage. These authors suggest that a recovery period of 2 to 3 estrous cycles is necessary to repair the damage caused by high summer temperatures and to enable the formation of functional oocytes for the subsequent breeding season.

The reduction in pregnancy rates in dairy cows under heat stress is well documented in the literature [9,33,35]. According to Roth (2020) [9], heat stress reduces the intensity of estrus and the likelihood of maintaining a pregnancy. In Spain, a decrease in pregnancy rates during the hottest season of the year was reported, with rates dropping to 27% compared to 44% during the cooler season [37]. Bernabucci et al. (2010) [35] emphasize that, on average, conception rates are reduced by approximately 24% during the summer.

The combination of multiple strategies has proven effective in improving pregnancy rates in dairy cows during the hot seasons. Methods such as timed artificial insemination, administration of gonadotropin-releasing hormone (GnRH), exogenous progesterone supplementation, and animal cooling systems have demonstrated efficiency in enhancing the reproductive performance of dairy cows [9,38,39]. Estrus detection combined with timed artificial insemination has shown increased pregnancy rates in cows during the summer [40]. The administration of GnRH on the 5th day after artificial insemination in third-lactation cows improved pregnancy rates [38]. However, Roth (2020) [9] highlights that the optimal timing of GnRH administration relative to ovulation remains unclear due to discrepancies in the literature, with some studies showing a beneficial effect on conception rates and others reporting no effect. According to Roth (2020) [9], early-stage progesterone supplementation has the potential to enhance embryo survival and conception success. Exogenous progesterone supplementation is commonly administered using an intravaginal progesterone-releasing device (CIDR). Administering CIDR on the 4th day after artificial insemination had a significant effect during summer, achieving a pregnancy rate of 43% compared to later administration on the 5th or 6th days [39].

## 6. Heat Stress and Its Impact on Health

Heat stress directly impacts the health of dairy cows. Prolonged periods of heat stress can lead to hyperthermia, which increases the production of reactive oxygen species and is associated with lymphocyte inhibition [6,41]. Dairy cows under thermal stress are more prone to diseases such as mastitis, metritis, lameness, and hepatic lipolysis [6]. The effects of heat stress, culminating in hyperthermia, initiate a pathogenic progression that begins with behavioral changes, including increased water intake and reduced dry matter intake, along with alterations in physiological responses, as previously discussed [7]. Burhans et al. (2022) [7] further report electrolyte imbalances, including acid-base disruptions, respiratory alkalosis with metabolic acidosis, and disturbances in sodium homeostasis.

Rising air temperatures affect animal behavior as cows attempt to enhance heat dissipation. To increase the surface area for heat exchange, cows spend more time standing, which may contribute to the increased incidence of lameness in dairy cows [6]. The prevalence of lameness is also observed to rise during summer when ambient temperatures are relatively higher [42]. Sanders et al. (2009) [42] additionally highlight that humid conditions lead to a greater occurrence of thin soles and hoof ulcers. Uterine diseases are also associated with heat stress in dairy cows, adversely affecting reproductive success. The incidence of metritis can increase during periods of heat stress [6].

Mastitis is one of the primary causes of increased somatic cell count (SCC) in milk, which decreases the quality of the product [43,44,45]. Heat stress poses an increased risk for the incidence of mastitis in dairy cows, as it depresses the immune response [6]. While somatic cell count tends to increase with the age of the cow, heat stress exacerbates this process. Studies conducted in Canada show higher SCC in the summer compared to other seasons of the year [46]. The risk of mastitis infections in the udder during thermal stress may also be linked to an increase in pathogens in the production environment. Bacteria such as Escherichia coli and Klebsiella pneumoniae do not survive in cold environments but are capable of surviving in conditions of elevated temperatures [6]. Therefore, coliform counts in bedding tend to increase during the summer, correlating with intramammary infections caused by coliforms [6].

## 7. Machine Learning for Predicting Heat Stress

Identifying heat stress in dairy cows can be a challenging task. Therefore, the use of behavioral and physiological parameters for identification is commonly employed, requiring a trained professional to recognize the symptoms. In this regard, several indices based on environmental variables have been developed to facilitate this identification, such as the Temperature and Humidity Index (THI) [10], the Black Globe Temperature and Humidity Index (BGHI) [47], Effective Temperature [1], among others. An alternative to simplify the process of identifying or even predicting heat stress in cows is the use of machine learning tools for thermal stress prediction. [13,48,49]

One approach to predicting heat stress in dairy cows is the use of machine learning techniques, which offer significant advantages over traditional indices like THI. While indices such as THI simplify the evaluation of thermal environments by combining variables like temperature and humidity, they fail to account for specific animal characteristics, such as physiological or behavioral responses. This limitation can result in less accurate estimates of heat’s impact on individual animals. In contrast, machine learning models integrate environmental data (e.g., air temperature, relative humidity, solar radiation, and wind speed) with physiological (e.g., respiratory rate, body temperature) and behavioral (e.g., rumination time, standing time) indicators, allowing for a more comprehensive and tailored analysis [12,50,51]. By collecting and preprocessing reliable data, algorithms such as artificial neural networks, support vector machines, and decision tree-based models can be trained to detect complex patterns that traditional indices cannot capture. To effectively use this tool, several studies [13,48,49] have been developed employing environmental variables and physiological and/or behavioral variables as input parameters for the model to infer the animal’s condition (Table 1).

To predict heat stress in Holstein cows, Pacheco et al. (2020) [13] developed machine learning models using climatic variables and physiological parameters, such as rectal temperature, respiratory rate, and surface temperature. The results showed that neural network models achieved an accuracy of 83% for respiratory rate and 84% for rectal temperature, significantly outperforming classical indices like THI and BGHI, which showed accuracies of only 68% and 55%, respectively. Becker et al. (2021) [48] applied machine learning algorithms to predict heat stress in 27 cows subjected to three different treatments: shade, sprinkler, and control. Their findings revealed that the Random Forest-based model was the most effective for predicting heat stress in cows under sprinkler treatment, while logistic regression performed better for cows in the control and shade groups. These studies highlight the increasing effectiveness of machine learning-based models compared to traditional approaches, such as climate indices, for providing more personalized and accurate predictions of heat stress.

These advancements are further supported by research from other authors exploring diverse modeling approaches [14,49]. Gorczyca and Gebremedhin (2020) [49] compared several algorithms, including Random Forest and Neural Networks, and found that these models offered greater accuracy in predicting physiological responses, such as respiratory rate and vaginal temperature, compared to traditional techniques, with ambient temperature emerging as the most impactful variable. Chung et al. [14] used a recurrent neural network (RNN) model to predict vaginal temperature from subdermal temperature data, demonstrating that RNNs outperformed linear regression models for short-term predictions. Although bioimplant-based monitoring solutions face challenges related to device size and battery life, they represent a promising step forward in real-time heat stress prediction, complementing prior models through continuous monitoring. These contributions underscore the ongoing evolution of heat stress prediction, emphasizing the importance of dynamic and individualized techniques.

Machine learning tools have also been used for classifying milk production, milk quality, as well as for establishing thermal stress thresholds based on milk production data [54]. Ji et al. (2020) [55] established dynamic heat stress limits in dairy cows, with data collected over five years from a farm with robotic milking, including milk production, milk temperature, and environmental data. The authors employed a model based on the Random Forest algorithm to categorize animals by age, body mass, and days of milk production. From the environmental data, the models were able to establish new thermal stress limits when compared to traditionally used thresholds, such as the THI.

The use of machine learning to predict heat stress in dairy cows offers several advantages over traditional methods. By integrating environmental, physiological, and behavioral data, machine learning techniques provide a more accurate and personalized analysis of each animal’s thermal conditions. Models such as neural networks, support vector machines, and Random Forest have demonstrated superior efficiency in predicting parameters like respiratory rate and rectal temperature. For example, Pacheco et al. (2020) [13] reported accuracies of up to 84%, surpassing the predictions provided by classical indices. Additionally, these tools can be applied to real-time monitoring, enabling immediate adjustments to management practices to optimize animal welfare and productivity.

However, the application of machine learning also presents challenges and limitations. Collecting large volumes of environmental and physiological data consistently and accurately requires advanced infrastructure and technical expertise among professionals. Ensuring data quality is crucial, as failures in data collection can negatively impact model performance [56]. Furthermore, while models like neural networks and Random Forest are highly effective, their complexity can pose challenges for farms that lack the resources to implement such sophisticated systems. The reliance on sensors and bioimplants, as demonstrated in the study by Chung et al. (2020) [14], also brings challenges regarding cost, maintenance, device size, and battery life. Despite these obstacles, integrating machine learning techniques represents a significant advancement, offering continuous and dynamic assessments of thermal conditions with more personalized and adaptive outcomes. The effectiveness of these tools can be further enhanced through targeted strategies to mitigate heat stress in dairy cows, promoting improved welfare and greater productive efficiency.

## 8. Strategies to Minimize Heat Stress

Various strategies are employed worldwide to reduce heat stress in dairy cows. These strategies vary depending on the local climate and the structural characteristics of the facilities [1,15,57,58,59,60,61,62]. The roofing of facilities used for housing milk-producing cows plays a key role in reducing the thermal radiation load reaching the animals [1,16]. In conjunction with the roof, the proper use of a well-designed ridge vent or roof vent promotes natural thermal ventilation [63,64]. In these facilities, an efficient ventilation system enhances heat exchange between the animal and the environment by maximizing convective heat transfer. Additionally, the use of evaporative cooling systems is common, particularly during the hottest hours of the day [57,65].

In animal production facilities, the ventilation system plays a crucial role in ensuring constant air renewal. This air exchange allows for the removal of hot air, humidity, and gases produced by the animals, replacing them with fresh air [1,15,16,66]. Additionally, ventilation significantly enhances thermal exchanges between the animal and the environment, aiding in heat dissipation. However, ventilation alone is not sufficient to reduce the air temperature inside the facility and ensure that the cow remains thermally comfortable. During the hottest hours of the day, especially in summer, air temperatures can become high, preventing the ventilation system from effectively maximizing heat exchange between the animal and the environment. A widely used strategy for cooling the environment and animals is evaporative adiabatic cooling [57,65,67]. As mentioned earlier, homeothermic animals, when experiencing thermal stress, primarily rely on sensible heat exchange to dissipate heat. When temperatures rise above certain limits, evaporative mechanisms are activated. In this context, the use of evaporative cooling in dairy cow production facilities is a strategy widely adopted in various regions of the world.

In an evaporative cooling system, methods such as misting or spraying are used within the facility, or even the use of evaporative panels, particularly when the facility is closed with tunnel ventilation systems [57,58,60,67]. These cooling methods can help keep the animal within the comfort zone or reduce thermal stress during the hottest hours of the day, without negatively affecting milk production or animal health [58,60,68].

In an experiment conducted in a tropical climate region of Brazil, in Pirassununga, São Paulo, Titto et al. (2013) [58] compared two ventilation systems across all four seasons and their impacts on milk production and cortisol concentration in the blood of lactating Holstein cows. The ventilation systems used were one with fans and misting nozzles, and another with natural ventilation. As a result, the authors observed that cows kept in environments with the misting ventilation system had higher milk production during spring and summer compared to cows kept without natural ventilation, showing a smaller seasonal variation in milk production. In another experiment, Dikmen et al. (2020) [69] assessed the thermal conditions of Free Stall-type facilities for lactating Holstein cows maintained in environments with positive pressure ventilation with misting nozzles and negative pressure ventilation with an evaporative adiabatic cooling system, analyzing its relation to rectal temperature and milk production during the summer and winter in Florida. The authors found that the seasonal variation in milk production and rectal temperature was smaller in facilities using the negative pressure ventilation system compared to those with positive pressure ventilation and misting nozzles.

However, the evaporative cooling system must be used with caution. It is most efficient in regions with high air temperatures and low relative humidity, due to the wet bulb depression, which is the difference between the dry bulb and wet bulb temperatures [1,15]. A greater wet bulb depression leads to a higher potential for evaporative cooling. Another factor to consider is the type of facility, as Compost Barn-type facilities, which have bedding undergoing semi-composting processes [20], may limit the use of this system. This is because increased bedding moisture can occur, impacting hygiene scores and potentially increasing the incidence of SCC in milk. An alternative in Compost Barn systems is the installation of sprinklers in the feeding alley and/or the waiting room for milking [68,70].

Chen et al. (2016) [70] conducted a study during the summer in California, comparing two different flow rates (1.3 and 4.9 L/min) installed in the feeding alley, with a treatment without sprinklers in a Free Stall-type facility. The results showed that the cows responded similarly to the different flow rates. It was observed that, regarding body temperature, in the sprinkler treatments, the temperature was lower by approximately 0.3 °C to 0.7 °C compared to the control during the hottest hours of the day. The authors also observed a higher average milk production in the sprinkler treatments, approximately 3.3 to 3.7 kg/day more than the control. They emphasized that the 1.3 L/min flow rate was more efficient in cooling compared to 4.9 L/min, as the lower flow rate resulted in similar body temperatures and milk production compared to the treatment without sprinklers. However, the authors also observed that in the sprinkler treatments, cows reduced their visits to the feeding alley.

Identifying heat stress in dairy cows is essential for efficient health and milk production management, as this factor directly affects animal physiology and milk yield. Understanding physiological parameters, such as rectal temperature and respiratory rate, alongside monitoring environmental variables, enables the implementation of effective mitigation strategies, such as ventilation systems, evaporative cooling, and facility adaptation. Combining these solutions, tailored to climatic conditions and structural characteristics, can help minimize the effects of heat, enhance animal welfare, and optimize productivity. Ongoing research and the refinement of these techniques are crucial to ensuring a favorable environment for cows, contributing to sustainable milk production.

## 9. Final Considerations

This review article focuses on thermoregulation in dairy cows, the physiological changes that occur when animals are exposed to heat-stressed environments, and the impacts on their health, productivity, and reproduction. It also discusses indicators of the thermal environment and the necessity of using techniques such as machine learning to predict heat stress in dairy cows. Additionally, it explores strategies for modifying the environment to mitigate heat stress.

Heat stress affects the health, milk production, and reproduction of dairy cows. These animals, of European origin, have relatively narrow thermal stress thresholds. To dissipate heat from the body core, various physiological and behavioral modifications occur. Respiratory rate tends to increase to maximize latent heat exchange, while heart rate also rises to intensify blood flow under the animal’s skin, along with behavioral modifications such as standing to maximize convective heat exchange. Furthermore, lactating cows experience a decrease in milk production when they remain under heat stress. Physiological and hormonal changes also influence the duration of estrus and embryonic development. Pathologies such as mastitis, metritis, and lameness are also consequences of thermally unfavorable environments for dairy cows.

Advances in machine learning techniques have been made to identify heat stress in dairy cows by correlating environmental and physiological variables, allowing for the prediction of the animal’s stress condition. Accurate models require large volumes of environmental, physiological, and behavioral data, which poses a challenge due to the difficulties in collecting data directly from the animals. One solution to improve these data collections and enhance model accuracy is the implementation of real-time acquisition systems and the analysis of thermal and behavioral images, which generate additional data for training. The use of these technologies holds great potential for the future of heat stress prediction, as they enable more effective and personalized data collection while accounting for individual variations. With continuous advancements in these techniques, heat stress can be predicted more efficiently, allowing for early interventions that promote animal welfare and optimize milk production. By combining traditional methods with these new approaches, the response to heat stress is strengthened, providing a healthier and more sustainable environment for dairy production.

Thus, cooling strategies must be implemented to reduce heat stress in dairy cows. The use of evaporative systems is effective in lowering air temperature and maximizing thermal exchanges for the animals. Evaporative cooling systems can be adopted in facilities with either positive or negative pressure ventilation. The use of evaporative panels in facilities with negative pressure ventilation significantly reduces air temperature, creating a thermally favorable environment. In open facilities, the use of sprinklers or misting nozzles in conjunction with fans proves to be effective in reducing thermal stress in dairy cows, ensuring animal welfare, milk production, and overall health.

## Figures and Tables

**Table 1:** Description of some studies carried out using machine learning to predict heat stress in dairy cows.

Reference	Description	Parameters	Models and Metrics *
Inadagbo et al. (2024) [11]	Computer vision models can be employed to predict animal behavior relevant to thermoregulation, using 96 animals with video collection over 45 days.	Behavioral: Drinking water; Brush use.	YoloV8 Accuracies: 96%
			Convulational neural network Accuracies: 93%
Li et al. (2024) [52]	Machine learning models can be used to predict core body temperature by analyzing data collected from 826 animals during 120 days, monitored on non-consecutive days, totaling 30 days of observation.	Air temperature; Relative humidity; Black globe temperature; Wind speed; Radiation intensity; THI; ITGU; Equivalent temperature; Latent and sensible heat exchange; Surface temperature; Rectal temperature.	Decision Tree/GWO–XGBoost R^2^ = 0.539, MAE = 0.232 °C, RMSE = 0.295 °C
Yan et al. (2024) [53]	Prediction of respiration rate in dairy cows by analyzing data collected from 826 cows monitored over 120 days.	Air temperature; Relative humidity; Black globe temperature; Airflow velocity; Solar radiation; Milk production; Respiration rate.	Decision Tree/CATBOOST R^2^ = 0.676 e RMSE = 9.341 breath/minute
Stygar et al. (2023) [51]	Dairy cow welfare classification based on sensors and farm records, using 318 cows from six farms over 135 days.	Accelerometers; Milk production; Lactation days; Lactation number; Welfare Quality^®^ (WQ^®^) protocol.	Decision Tree/XGBoost Sensitivity: 0.44 specificity: 0.68
Brezov et al. (2023) [12]	Prediction of rectal temperature of dairy cows, with 295 animals, during 120 days.	Rectal temperature; Respiratory rate; Heart rate; Thermal imaging; THI.	Recurrent neural network R^2^ = 0.73 MAE = 0.1 °C
Bovo et al. (2021) [50]	Evaluation of trends in daily milk production of a cow in relation to environmental conditions, using 91 dairy cows, over two years.	Air temperature; Relative humidity; THI; Milk production; Milk days.	Random Forest Total forecast error: 2%
Becker et al. (2021) [48]	Classification of heat stress in dairy cows, using 27 cows, monitored for 60 days.	THI; Ruminal temperature; Hygiene score; Activity; Respiratory rate.	Logistic regression R^2^ = 0.53
			Random Forest R^2^ = 0.97
Fuentes et al. (2020) [54]	Modeling of milk productivity and quality based on cow and environmental data, using 348 cows divided into two groups, for 2 years 10 months.	Air temperature; Relative humidity; THI; Lactation days; Lactation number; Milking frequency; Milk production; Milk protein; Milk fat; Somatic cells; Live weight.	Neural network MSE = 0.0189 and 0.0157 kg/animal, for each group of animals.
Gorczyca et al. (2020) [49]	Classification of thermal stressors for dairy cows, using 19 dairy cows, monitored for 21 days.	Air temperature; Relative humidity; Solar radiation; Air velocity; Respiration rate; Skin temperature; Vaginal temperature.	Random Forest RMSE = 9.695 breath/minute RMSE = 0.434 °C skin temperature.
			Neural networks RMSE = 0.434 °C vaginal temperature.

* RMSE is Root mean square error; MAE is Mean absolute error; MSE is Mean square error.

## Data Availability

No data were used for the research described in the article.
